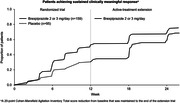# Sustained clinically meaningful response in patients with agitation associated with dementia due to Alzheimer’s disease treated with brexpiprazole: *post hoc* analysis

**DOI:** 10.1002/alz.092315

**Published:** 2025-01-03

**Authors:** Malaak Brubaker, David Wang, Sanjeda R Chumki, Pedro Such, Zhen Zhang, Anton M Palma

**Affiliations:** ^1^ Otsuka Pharmaceutical Development & Commercialization Inc., Princeton, NJ USA; ^2^ Lundbeck LLC, Deerfield, IL USA; ^3^ H. Lundbeck A/S, Valby, Copenhagen Denmark

## Abstract

**Background:**

A reduction in the frequency of agitation behaviors is a clinically meaningful outcome among patients with agitation associated with dementia due to Alzheimer’s disease. This *post hoc* analysis aimed to determine the percentage of patients treated with brexpiprazole who achieved sustained clinically meaningful response (CMR), over 12 and 24 weeks.

**Method:**

Data for brexpiprazole 2 or 3 mg/day were obtained from two trials of patients with agitation associated with dementia due to Alzheimer’s disease: a 12‐week, randomized, double‐blind, placebo‐controlled trial (ClinicalTrials.gov identifier: NCT03548584) and a 12‐week, active‐treatment extension trial (NCT03594123). According to previous anchor‐ and distribution‐based analyses, a 20‐point reduction from baseline in Cohen‐Mansfield Agitation Inventory Total score represents a meaningful within‐patient change in this population. Hazard ratios (HRs) for non‐response and Kaplan–Meier curves for the cumulative proportions achieving CMR (a 20‐point score reduction) and sustained clinically meaningful response (SCMR; a 20‐point score reduction that was maintained to trial end) were calculated over (A) 12 weeks (data from the randomized trial for brexpiprazole versus placebo) and (B) 24 weeks (data from the randomized + extension trials for ‘prior brexpiprazole’ [i.e., received brexpiprazole in both trials] versus ‘prior placebo’ [i.e., received placebo in the randomized trial and brexpiprazole in the extension trial]).

**Result:**

Over 12 weeks, the percentages of patients achieving CMR were 61.8% for brexpiprazole (n = 225) versus 44.8% for placebo (n = 116); HR = 0.64; p = 0.006. Corresponding percentages of patients achieving SCMR were 49.3% for brexpiprazole versus 32.8% for placebo; HR = 0.58; p = 0.004. Over 24 weeks, the percentages of patients achieving CMR were 81.8% for prior brexpiprazole (n = 159) versus 72.6% for prior placebo (n = 95); HR = 0.63; p = 0.011. Corresponding percentages of patients achieving SCMR were 75.5% for prior brexpiprazole versus 68.4% for prior placebo; HR = 0.59; p = 0.010 (Figure).

**Conclusion:**

Among patients with agitation associated with dementia due to Alzheimer’s disease, a high percentage of patients on brexpiprazole 2 or 3 mg/day achieved sustained clinically meaningful response.